# 3-(4-Bromo­phen­yl)-*N*,*N*-dimethyl-3-oxopropan-1-aminium chloride

**DOI:** 10.1107/S1600536811041985

**Published:** 2011-10-22

**Authors:** Rodrigo Abonia, Dieter Schollmeyer, Danny Arteaga

**Affiliations:** a Departamento de Química, Universidad del Valle, AA 25360 Cali, Colombia; b University Mainz, Duesbergweg 10-14, 55099 Mainz, Germany

## Abstract

The title compound, C_11_H_15_BrNO^+^·Cl^−^, was obtained as a precursor within our current program for the synthesis of new β-amino­alcohols *via* a Mannich-type reaction. The protonated amino N atom is hydrogen bonded to the chloride anion. With exception of one methyl group, the cation is approximately planar (r.m.s. deviation for all non H-atoms = 0.069 Å).

## Related literature

For (*N*,*N*-dialkyl­amino)­propiophenones, see: Alper *et al.* (2002 ▶); Pupo *et al.* (2003 ▶); Abonia *et al.* (2004 ▶). For details of the synthesis, see: Brandes & Roth (1967 ▶); Vogel *et al.* (1978 ▶).
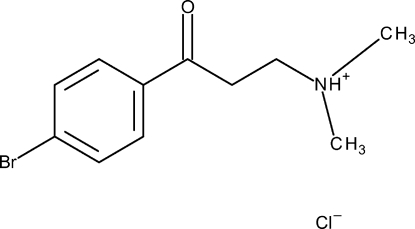


## Experimental

### 

#### Crystal data

C_11_H_15_BrNO^+^·Cl^−^
*M* *_r_* = 292.60
Monoclinic, 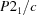

*a* = 10.5050 (8) Å
*b* = 12.5694 (5) Å
*c* = 10.6483 (5) Å
β = 115.594 (2)°
*V* = 1268.06 (12) Å^3^
*Z* = 4
Cu *K*α radiation
μ = 6.16 mm^−1^
*T* = 295 K
0.44 × 0.26 × 0.26 mm

#### Data collection

Enraf–Nonius CAD-4 diffractometer
Absorption correction: ψ scan (*CORINC*; Dräger & Gattow, 1971 ▶) *T*_min_ = 0.61, *T*_max_ = 1.00
2564 measured reflections
2564 independent reflections
2258 reflections with *I* > 2σ(*I*)
3 standard reflections every 60 min  intensity decay: 5%

#### Refinement

*R*[*F* ^2^ > 2σ(*F* ^2^)] = 0.045
*wR*(*F* ^2^) = 0.123
*S* = 1.12
2564 reflections
146 parameters
Only H-atom displacement parameters refined
Δρ_max_ = 0.72 e Å^−3^
Δρ_min_ = −0.65 e Å^−3^

### 

Data collection: *CAD-4 Software* (Enraf–Nonius, 1989 ▶); cell refinement: *CAD-4 Software*; data reduction: *CORINC* (Dräger & Gattow, 1971 ▶); program(s) used to solve structure: *SIR97* (Altomare *et al.*, 1999 ▶); program(s) used to refine structure: *SHELXL97* (Sheldrick, 2008 ▶); molecular graphics: *PLATON* (Spek, 2009 ▶); software used to prepare material for publication: *PLATON*.

## Supplementary Material

Crystal structure: contains datablock(s) I, global. DOI: 10.1107/S1600536811041985/bt5672sup1.cif

Structure factors: contains datablock(s) I. DOI: 10.1107/S1600536811041985/bt5672Isup2.hkl

Supplementary material file. DOI: 10.1107/S1600536811041985/bt5672Isup3.cml

Additional supplementary materials:  crystallographic information; 3D view; checkCIF report

## Figures and Tables

**Table 1:** Hydrogen-bond geometry (Å, °)

*D*—H⋯*A*	*D*—H	H⋯*A*	*D*⋯*A*	*D*—H⋯*A*
N10—H10⋯Cl1	1.00	1.99	2.983 (2)	171

**Fig. 1.**
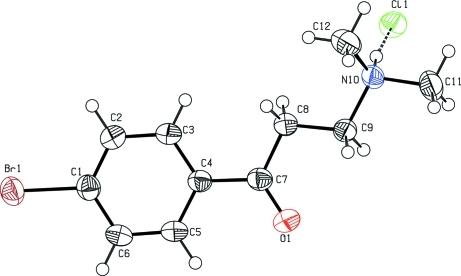
View of compound I. Displacement ellipsoids are drawn at the 50% probability level.

## supplementary crystallographic information

### Comment

The classical method for the synthesis of 3-(*N,N*-dialkylamino)propiophenone salts is the well known Mannich reaction between an alkyl aryl ketone, dialkylamine hydrochloride and polyformaldehyde in refluxing ethanol (Vogel *et al.*, 1978). In this approach, the *N,N*-dialkylmethyleneammonium chloride (H_2_C=NR_2_Cl) is formed *in situ*, which suffers a Michael type addition from the methylene active ketone to render the expected Mannich adduct (Brandes *et al.*, 1967).

The 3-(*N,N*-dialkylamino)propiophenone salts are visualized as synthetic equivalents of the less stable and more reactive α,β-unsaturated aryl vinyl ketones. For instance, they can react with nucleophiles like amines through a Michael type addition which could lead to the formation of β-aminoalcohols as is our purpose with compound (I).

In the crystal the title compound adopts an essentially planar structure with a dihedral angle of 5.0 (2)° between the almost planar aminopropane-1-one group (maximal deviation from least square plane 0.038Å at C9) and the phenyl ring. The protonated N10 atom forms a hydrogen bond to Cl1 (N10—H10···Cl1 1.99 Å).

### Experimental

A mixture of dimethylamine hydrochloride (2.0 g, 25 mmol), polyformaldehyde (0.754 g, 25 mmol), *p*-bromoacetophenone (3.66 g, 9.2 mmol), 95% ethanol (4 mL) and conc HCl (0.02 mL) was heated at reflux in an oil bath during 3 h (Vogel *et al.* (1978)). After complete disappearance of the starting acetophenone, as monitored by thin-layer chromatography, the hot mixture was filtered; acetone (15 mL) was added to the filtrate and cooled into the freezer overnight. The resulting solid was filtered, washed with acetone (2 *x* 5 mL) and dried at ambient temperature affording the title compound (I), as white solid [yield 94%, m.p. 495 K].

Crystals of (I) suitable for single-crystal X-ray diffraction were grown by slow evaporation at ambient temperature and in air, from a 1:1 ethanol:acetone solution.

### Refinement

Hydrogen atoms attached to carbons were placed at calculated positions with C—H = 0.95 Å (aromatic) or 0.98–0.99 Å (*sp*^3^ C-atom). All H atoms were refined in the riding-model approximation with isotropic displacement parameters.

### Crystal data

**Table:**

C_11_H_15_BrNO^+^·Cl^−^	*F*(000) = 592
*M**_r_* = 292.60	*D*_x_ = 1.533 Mg m^−^^3^
Monoclinic, *P*2_1_/*c*	Melting point: 495 K
Hall symbol: -P 2ybc	Cu *K*α radiation, λ = 1.54178 Å
*a* = 10.5050 (8) Å	Cell parameters from 25 reflections
*b* = 12.5694 (5) Å	θ = 64–74°
*c* = 10.6483 (5) Å	µ = 6.16 mm^−^^1^
β = 115.594 (2)°	*T* = 295 K
*V* = 1268.06 (12) Å^3^	Block, brown
*Z* = 4	0.44 × 0.26 × 0.26 mm

### Data collection

**Table:**

Enraf–Nonius CAD-4 diffractometer	2258 reflections with *I* > 2σ(*I*)
Radiation source: rotating anode	*R*_int_ = 0.000
graphite	θ_max_ = 73.8°, θ_min_ = 4.7°
θ/2ω scans	*h* = −11→13
Absorption correction: ψ scan (CORINC; Dräger & Gattow, 1971)	*k* = −15→0
*T*_min_ = 0.61, *T*_max_ = 1.00	*l* = −13→0
2564 measured reflections	3 standard reflections every 60 min
2564 independent reflections	intensity decay: 5%

### Refinement

**Table:**

Refinement on *F*^2^	Secondary atom site location: structure-invariant direct methods
Least-squares matrix: full	Hydrogen site location: difference Fourier map
*R*[*F*^2^ > 2σ(*F*^2^)] = 0.045	Only H-atom displacement parameters refined
*wR*(*F*^2^) = 0.123	*w* = 1/[σ^2^(*F*_o_^2^) + (0.0762*P*)^2^ + 0.3958*P*] where *P* = (*F*_o_^2^ + 2*F*_c_^2^)/3
*S* = 1.12	(Δ/σ)_max_ = 0.001
2564 reflections	Δρ_max_ = 0.72 e Å^−^^3^
146 parameters	Δρ_min_ = −0.65 e Å^−^^3^
0 restraints	Extinction correction: *SHELXL97* (Sheldrick, 2008), Fc^*^=kFc[1+0.001xFc^2^λ^3^/sin(2θ)]^-1/4^
Primary atom site location: structure-invariant direct methods	Extinction coefficient: 0.0047 (5)

### Special details

**Table:**

Experimental. IR (KBr disk): 3080, 3024, 2954, 2912, 2628, 2543 (br), 2503, 2440 (br), 1684 (C=O), 1580, 1473, 1391, 1329, 1216, 1065, 1002, 963, 787 cm^-1. 1^H-NMR (DMSO-d6): 2.79 (s, 6H), 3.39 (t, J = 7.5 Hz, 2H), 3.64 (t, J = 7.2 Hz,2*H*), 7.78 ("d", J =8.4 Hz, 2H), 7.95 ("d", J = 8.4 Hz, 2H), 10.93 (bs, 1H, NH) p.p.m.; ^13^C-NMR (DMSO-d6): 33.2, 42.1, 51.5, 127.8, 130.0, 131.9, 134.9, 196.0 (C=O) p.p.m..
Geometry. All e.s.d.'s (except the e.s.d. in the dihedral angle between two l.s. planes) are estimated using the full covariance matrix. The cell e.s.d.'s are taken into account individually in the estimation of e.s.d.'s in distances, angles and torsion angles; correlations between e.s.d.'s in cell parameters are only used when they are defined by crystal symmetry. An approximate (isotropic) treatment of cell e.s.d.'s is used for estimating e.s.d.'s involving l.s. planes.
Refinement. Refinement of *F*^2^ against ALL reflections. The weighted *R*-factor *wR* and goodness of fit *S* are based on *F*^2^, conventional *R*-factors *R* are based on *F*, with *F* set to zero for negative *F*^2^. The threshold expression of *F*^2^ > σ(*F*^2^) is used only for calculating *R*-factors(gt) *etc*. and is not relevant to the choice of reflections for refinement. *R*-factors based on *F*^2^ are statistically about twice as large as those based on *F*, and *R*- factors based on ALL data will be even larger.

### Fractional atomic coordinates and isotropic or equivalent isotropic displacement parameters (Å^2^)

**Table:**

	*x*	*y*	*z*	*U*_iso_*/*U*_eq_	
Br1	0.31084 (4)	0.13998 (3)	0.50877 (3)	0.04655 (18)	
Cl1	0.89690 (9)	−0.34411 (6)	1.32816 (9)	0.0430 (2)	
O1	0.7418 (3)	0.0972 (2)	1.2059 (2)	0.0539 (6)	
C1	0.4405 (3)	0.1090 (2)	0.6944 (3)	0.0374 (6)	
C2	0.5264 (3)	0.0213 (3)	0.7200 (3)	0.0436 (7)	
H2	0.5200	−0.0221	0.6467	0.058 (11)*	
C3	0.6223 (3)	−0.0025 (3)	0.8549 (3)	0.0411 (7)	
H3	0.6794	−0.0623	0.8722	0.040 (9)*	
C4	0.6335 (3)	0.0629 (2)	0.9646 (3)	0.0335 (6)	
C5	0.5434 (4)	0.1497 (2)	0.9364 (4)	0.0419 (7)	
H5	0.5478	0.1922	1.0097	0.060 (11)*	
C6	0.4478 (4)	0.1745 (3)	0.8029 (3)	0.0430 (7)	
H6	0.3893	0.2336	0.7854	0.054 (11)*	
C7	0.7377 (3)	0.0416 (2)	1.1111 (3)	0.0362 (6)	
C8	0.8395 (3)	−0.0495 (2)	1.1373 (3)	0.0369 (6)	
H8A	0.7874	−0.1160	1.1125	0.058 (8)*	
H8B	0.8885	−0.0414	1.0788	0.058 (8)*	
C9	0.9456 (3)	−0.0535 (2)	1.2878 (3)	0.0360 (6)	
H9A	0.9986	0.0126	1.3113	0.046 (7)*	
H9B	0.8957	−0.0590	1.3459	0.046 (7)*	
N10	1.0466 (3)	−0.14452 (17)	1.3199 (3)	0.0358 (6)	
H10	0.9959	−0.2136	1.3123	0.053 (11)*	
C11	1.1447 (5)	−0.1452 (3)	1.4695 (4)	0.0578 (11)	
H11A	1.2084	−0.2042	1.4889	0.082 (10)*	
H11B	1.0919	−0.1518	1.5236	0.082 (10)*	
H11C	1.1974	−0.0800	1.4932	0.082 (10)*	
C12	1.1245 (4)	−0.1462 (3)	1.2322 (4)	0.0526 (9)	
H12A	1.0585	−0.1462	1.1357	0.101 (11)*	
H12B	1.1817	−0.2092	1.2524	0.101 (11)*	
H12C	1.1838	−0.0845	1.2518	0.101 (11)*	

### Atomic displacement parameters (Å^2^)

**Table:**

	*U*^11^	*U*^22^	*U*^33^	*U*^12^	*U*^13^	*U*^23^
Br1	0.0519 (3)	0.0423 (2)	0.0400 (2)	0.00146 (13)	0.01472 (18)	0.00820 (13)
Cl1	0.0541 (5)	0.0294 (4)	0.0461 (4)	−0.0091 (3)	0.0223 (4)	−0.0025 (3)
O1	0.0644 (15)	0.0500 (14)	0.0408 (12)	0.0130 (12)	0.0166 (12)	−0.0113 (11)
C1	0.0398 (15)	0.0351 (15)	0.0376 (15)	−0.0020 (12)	0.0170 (13)	0.0041 (12)
C2	0.0492 (18)	0.0441 (18)	0.0381 (15)	0.0058 (14)	0.0193 (14)	−0.0037 (13)
C3	0.0436 (16)	0.0380 (16)	0.0418 (16)	0.0102 (13)	0.0187 (14)	−0.0025 (13)
C4	0.0364 (14)	0.0294 (14)	0.0363 (14)	0.0003 (11)	0.0170 (12)	−0.0011 (11)
C5	0.0505 (18)	0.0309 (16)	0.0444 (18)	0.0058 (12)	0.0206 (15)	−0.0055 (12)
C6	0.0519 (19)	0.0291 (15)	0.0471 (18)	0.0082 (13)	0.0206 (15)	−0.0004 (13)
C7	0.0401 (15)	0.0284 (13)	0.0406 (15)	−0.0010 (11)	0.0181 (13)	−0.0009 (12)
C8	0.0418 (15)	0.0318 (15)	0.0347 (14)	0.0019 (12)	0.0142 (12)	−0.0018 (11)
C9	0.0467 (16)	0.0270 (14)	0.0339 (14)	−0.0007 (12)	0.0170 (13)	0.0003 (11)
N10	0.0458 (14)	0.0228 (11)	0.0342 (13)	−0.0019 (9)	0.0130 (11)	0.0029 (9)
C11	0.070 (2)	0.0371 (19)	0.0411 (19)	0.0028 (16)	0.0002 (18)	0.0031 (14)
C12	0.057 (2)	0.044 (2)	0.063 (2)	0.0121 (15)	0.0319 (19)	0.0101 (16)

### Geometric parameters (Å, °)

**Table:**

Br1—C1	1.894 (3)	C2—H2	0.9300
O1—C7	1.213 (4)	C3—H3	0.9300
C1—C2	1.375 (4)	C5—H5	0.9300
C1—C6	1.394 (4)	C6—H6	0.9300
C2—C3	1.385 (4)	C8—H8A	0.9700
C3—C4	1.391 (4)	C8—H8B	0.9700
C4—C5	1.390 (4)	C9—H9A	0.9700
C4—C7	1.493 (4)	C9—H9B	0.9700
C5—C6	1.376 (5)	C11—H11A	0.9600
C7—C8	1.509 (4)	C11—H11B	0.9600
C8—C9	1.507 (4)	C11—H11C	0.9600
C9—N10	1.496 (4)	C12—H12A	0.9600
N10—C11	1.477 (4)	C12—H12B	0.9600
N10—C12	1.484 (5)	C12—H12C	0.9600
N10—H10	1.0000		
			
C2—C1—C6	120.9 (3)	C1—C6—H6	121.00
C2—C1—Br1	119.1 (2)	C5—C6—H6	121.00
C6—C1—Br1	120.0 (2)	C7—C8—H8A	109.00
C1—C2—C3	120.0 (3)	C7—C8—H8B	109.00
C2—C3—C4	120.2 (3)	C9—C8—H8A	109.00
C5—C4—C3	118.7 (3)	C9—C8—H8B	109.00
C5—C4—C7	119.3 (3)	H8A—C8—H8B	108.00
C3—C4—C7	122.0 (3)	N10—C9—H9A	109.00
C6—C5—C4	121.7 (3)	N10—C9—H9B	109.00
C5—C6—C1	118.5 (3)	C8—C9—H9A	109.00
O1—C7—C4	120.9 (3)	C8—C9—H9B	109.00
O1—C7—C8	121.1 (3)	H9A—C9—H9B	108.00
C4—C7—C8	118.0 (2)	N10—C11—H11A	109.00
C9—C8—C7	111.2 (2)	N10—C11—H11B	109.00
N10—C9—C8	113.2 (2)	N10—C11—H11C	109.00
C11—N10—C12	111.2 (3)	H11A—C11—H11B	109.00
C11—N10—C9	110.2 (2)	H11A—C11—H11C	110.00
C12—N10—C9	113.4 (2)	H11B—C11—H11C	109.00
C1—C2—H2	120.00	N10—C12—H12A	110.00
C3—C2—H2	120.00	N10—C12—H12B	109.00
C2—C3—H3	120.00	N10—C12—H12C	110.00
C4—C3—H3	120.00	H12A—C12—H12B	109.00
C4—C5—H5	119.00	H12A—C12—H12C	109.00
C6—C5—H5	119.00	H12B—C12—H12C	109.00
			
C6—C1—C2—C3	−0.5 (5)	C5—C4—C7—O1	2.1 (5)
Br1—C1—C2—C3	179.7 (3)	C3—C4—C7—O1	−177.0 (3)
C1—C2—C3—C4	−0.8 (5)	C5—C4—C7—C8	−176.6 (3)
C2—C3—C4—C5	2.3 (5)	C3—C4—C7—C8	4.2 (4)
C2—C3—C4—C7	−178.6 (3)	O1—C7—C8—C9	−4.1 (4)
C3—C4—C5—C6	−2.5 (5)	C4—C7—C8—C9	174.6 (2)
C7—C4—C5—C6	178.4 (3)	C7—C8—C9—N10	178.4 (2)
C4—C5—C6—C1	1.2 (5)	C8—C9—N10—C11	−178.4 (3)
C2—C1—C6—C5	0.3 (5)	C8—C9—N10—C12	56.2 (4)
Br1—C1—C6—C5	−179.9 (2)		

### Hydrogen-bond geometry (Å, °)

**Table:**

*D*—H···*A*	*D*—H	H···*A*	*D*···*A*	*D*—H···*A*
N10—H10···Cl1	1.00	1.99	2.983 (2)	171
